# Supplementary material for: Chromothripsis during telomere crisis is independent of NHEJ, and consistent with a replicative origin
Source: Genome Res. 2019 May;29(5):737–49. doi: 10.1101/gr.240705.118 (PMC6499312; doi:10.1101/gr.240705.118)
Supplement: Supplemental Material [file supp_gr.240705.118_Supplemental_file_1.zip › contigs/annotated_contigs/DB91/contig.2.DB91_length_637_mean_cov_7.53532182104.docx]

**DB91_length_637_mean_cov_7.53532182104**

```
GGGCATTGCCACTTCTAAATCTGCAAGAGAACCAGAGGTTCGCATAACGTCATCAACAAGATCATGACATATGATGAGGCTATGCATAT
 >chr7:122331595-122331943 + E=1e-197
AGTCCTGCAGTGACACTATGAAAGTCCATTGTCACCCTCCCATGAAGGCAGACTGTTCCTGGCTCTCTGGAGCAATGTTGATTGAGAGG

AATGCATTGGTCAGGTCCACCACACAGTGGCACCATCCCAGTTCTATTGTCAAGCAGTCCAGCAAGTCCGTGACAGACAGCACAGCTGT

CAAGCTGTGCAAAACATCCACCCCCAGAATGTCTCCAGGCATGGGAGAGATGTGCACAGTGGACAAGCAGACAGCCAA|GTG|AGAGCT
                                                                               >chr7:12
GTGGATGGGAAAGCTGCTGAATAAAACTACATTTCACCTGCTTACGGCCCCGAGTGTTCTTTCAGCTATCTGTCCATCCACCCACTCCC
2332586-122332878 - E=6e-161
CTCGGACCTCAGCAGGGGCTGGACCCTGGGCATGACGTTTGGTGTACTCATGAACCTGACAGTTGGCATAGTTGGCCAGATGAGGTGAG

TGGGTCTTCAGTTCTGNGGGGTCTTGGGTCGGCTATGTGGCCGCAGCATGGGCTGTGGTACCCAGTGGCAGCGGTGCTGCCTGGGTGGG

CCCCAGTGGAAACGTG
```
